# Minor-but-Complex Liver Resection: An Alternative to Major Resections for Colorectal Liver Metastases Involving the Hepato-Caval Confluence

**DOI:** 10.1097/MD.0000000000001188

**Published:** 2015-07-24

**Authors:** Lucio Urbani, Gianluca Masi, Marco Puccini, Piero Colombatto, Caterina Vivaldi, Riccardo Balestri, Antonio Marioni, Valerio Prosperi, Francesco Forfori, Gabriella Licitra, Chiara Leoni, Adriana Paolicchi, Piero Boraschi, Alessandro Lunardi, Carlo Tascini, Maura Castagna, Piero Buccianti

**Affiliations:** From the General Surgery Unit (LU, MP, RB, AM, VP, PB); Oncology Unit (GM, CV); Hepatology Unit (PC); Anaesthesiology and Intensive Care Unit (FF, GL, CL, AP); Radiology Unit (PB, AL); Infectious Disease Unit (CT); Pathology Unit (MC); Azienda Ospedaliero-Universitaria Pisana, Ospedale Nuovo Santa Chiara, Cisanello, Pisa, Italy.

## Abstract

Major hepatectomy (MH) is often considered the only possible approach for colorectal liver metastasis (CRLM) at the hepato-caval confluence (CC), but it is associated with high morbidity and mortality. With the aim to reduce MH, we developed the “minor-*but*-complex” (M*b*C) technique, which consists in the resection of less than 3 adjacent liver segments with exposure of the CC and preservation of hepatic outflow until spontaneous maturation of peripheral intrahepatic shunts between main hepatic veins. We have evaluated applicability and outcome of M*b*C resections for the treatment of CRLM involving the CC.

In this retrospective cohort study, all consecutive liver resections (LR) performed for CRLM located in segments 1, 7, 8, or 4a were classified as MINOR – removal of <3 adjacent segments; M*b*C – removal of <3 adjacent segments with CC exposure; and MH – removal of ≥3 adjacent segments. The rate of avoided MH was obtained by the difference between the rate of potentially MH (PMH) plus potentially inoperable cases and the rate of the MH performed. Taking into account that postoperative mortality is mainly related to the amount of resected liver, M*b*C was compared with minor resections for safety, complexity, and outcome.

Of the 59 LR analyzed, 29 (49.1%) were deemed PMH and 4 (6.8%) potentially inoperable. Eventually, MH was performed only in 8 (13.5%) with a decrease rate of 42.4%. Minor LR was performed in 23 (39.0%) and M*b*C LR in 28 (47.5%) patients. Among M*b*C cases, 32.1% had previous liver treatments, 39.3% required vascular reconstruction (no reconstructed vessel thrombosis occurred before maturation of peripheral intrahepatic shunts between main hepatic veins), and 7.1% had grade IIIb–IV complications, their median hospital stay was 9 days and 90-day mortality was 0%. After a median follow-up of 22.2 months, oncological results were comparable with those of minor resections.

M*b*C hepatectomy lowers the need for MH and allows for the resection of potentially inoperable patients without negative impact on safety and survival.

## INTRODUCTION

Surgical resection of colorectal liver metastases (CRLM) is the only treatment associated with cure or long-term survival in this setting. Unfortunately, only a minority of patients with liver metastases are suitable for liver resection (LR). Therefore, strategies to increase the rate of resectable patients are under active investigation.

A major hepatectomy (MH) is commonly defined as the resection of 3 or more adjacent hepatic segments. The presence of a CRLM in contact with the right or middle hepatic vein (MHV) at the hepato-caval confluence (CC) is usually an indication to perform a major LR, since all the drained liver segments are removed. Due to increased rates of perioperative morbidity and mortality related to MH,^1^ a lot of strategies were developed in the last 20 years to increase the future liver remnant volume: portal vein embolization (PVE),^2^ portal vein ligation,^3^ two-stage hepatectomy,^4,5^ and more recently the novel “ALPPS” technique (Associating Liver Partition and Portal vein ligation for staged hepatectomy).^6,7^

In 2005 Torzilli et al proposed another approach to reduce the risk associated with MH introducing the concept of the “radical but conservative” ultrasonography-guided liver surgery. The basis of this approach is to overcome the limiting factor of the future liver remnant by transforming an MH in a minor hepatectomy.^8^ Torzilli et al for the first time studied the importance of the liver skeleton and the fundamental role of communicating veins between adjacent HV identified by the extensive use of intraoperative ultrasound (IOUS).^9^ The radical but conservative philosophy is a new way to think the liver surgery and prompted new techniques to spare liver parenchyma when HV should be resected at the CC,^10–12^ as an extension of the interventions proposed by Makuuchi et al based on the preservation of the inferior right hepatic vein (IRHV).^13^ All these procedures, however, are based on the capability to detect and preserve the communicating veins. This implies a high skill in performing IOUS and to turn it into a guide to safely resect the liver parenchyma.

Most liver surgeons are unable to detect intraoperativelly such small anastomoses and are reluctant to leave congested areas in the liver remnant. The same problem was encountered in living donor right liver transplantation where congested areas mean nonfunctional liver, but preservation of an adequate outflow allows for the subsequent development of those anastomoses.

Based on this concept, we have introduced the “minor-*but*-complex” (M*b*C) liver resection paradigm as an alternative to MH. It consists in maintaining the blood flow in the main intrahepatic vessels in contact with CRLM using surgical skills maturated in the field of liver transplantation, as a “bridge” to the peripheral intrahepatic shunts maturation. In this way, the parenchymal sparing liver surgery is not dependent on the extremely difficult ultrasound identification and preservation of the intrahepatic shunts between HV.

The aim of this study is to evaluate whether the M*b*C approach could reduce the need for MH for CRLM located in segments 1, 7, 8, or the cranial portion of segment 4 without affecting the clinical outcome. Therefore, surgical complexity, safety, and oncological results of the M*b*C approach was compared with those of minor hepatic resections performed for CRLM far from the CC.

## METHODS

### Terminology

**FIGURE 1:**
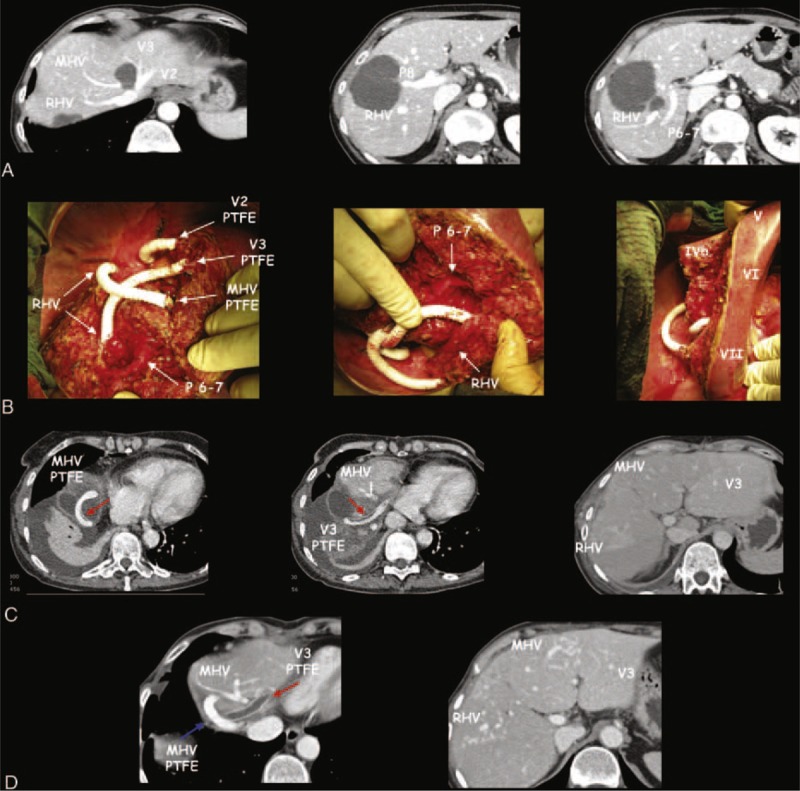
A case of minor-*but*-complex hepatic resection with hepatic vein reconstruction with ringed PTFE graft used as “a bridge” to intrahepatic shunt maturation. This patient had 6 synchronous colorectal metastasis (CRLM) and after the resection of primary tumor had good response to 8 cycles of FOLFOXIRI plus Bevacizumab. (A) This case was judged potentially inoperable on the basis of preoperative CT scan. Liver outflow was infiltrated by different CRLM at the hepato-caval confluence (CC) involving the left hepatic vein (HV) for segments 2 and 3 (V2, V3) and middle HV (MHV), and far from the CC involving the right HV (RHV). Liver inflow was also involved by CRLM interesting right portal branch for segment VIII (P8) and the CRLM infiltrating RHV close to the posterior right portal branch (P6–7). (B) The intraoperative field of the resection of segment VIII–IVa partially extended to segment VII and II and to the paracaval portion of segment I with the exposure of all the main intrahepatic vascular structures at the CC. Left hepatic outflow was reconstructed with 3 ringed PTFE grafts: V2 was reconstructed directly on the cava vein, V3 was reconstructed on the RHV where it was tangentially resected for the adjacent CRLM, the MHV was reconstructed at the level of the confluence of the hepatic vein of segment VII with the RHV. Right inflow was preserved with the complete exposure of P6–7 and with the section of P8 at his origin. The resected liver presented no sign of congestion with all the residual segments well perfused. The liver cut surface was 241 cm^2^, the cumulative clamping time was 292 minutes, 8 units of red packed cells were requested (preoperative anemia 8,6 Hb and metabolic acidosis due to chemotherapy), the postoperative course was characterized by transient ascites, and the patient was discharged on the 14th postoperative day; successively, a delayed biloma was treated with a percutaneous drainage. Eight months after liver surgery the patient is alive and well without any sign of disease recurrences. (C) The CT scan on the 11th postoperative day shows a well perfused left liver in spite of the thrombosis of the V2 PTFE graft (due to presence of intrahepatic shunts between V2 and V3). There were no-evident shunts between V3 to MHV and MHV to RHV. Red arrows indicate a laminar thrombus in both MHV and V3 PTFE. (D) The CT scan 5 months after shows the resolution of the thrombus in the MHV PTFE graft (blue arrow) and the complete thrombosis of the V3 PTFE graft (red arrow) with a well perfused left liver because of the spontaneous maturation of peripheral shunts between V3 and MHV; peripheral shunts are also maturating around RHV.

The terminology for liver anatomy and resections is based on Brisbane classification.^14^ Hepatic resections are considered major when at least 3 adjacent segments are removed. LRs requiring the exposure of inferior vena cava (IVC), the main trunks of the HV, or both are considered *complex*. “Potentially major hepatectomy” (PMH) is defined before LR when the preoperative-computed tomography (CT) scan showed a CRLM in contact with the right HV (RHV) and/or the middle HV (MHV) at the CC. The hepatectomy was not considered PMH when the preoperative-CT scan showed an IRHV allowing a safe RHV resection.^13^ Patients were defined potentially inoperable when, at the preoperative-CT scan, the CRLM appeared in contact with the right and left inflow and/or outflow requiring a left and a right hepatectomy at the same time, thus precluding also the treatment with PVE, two-stage resection and ALPPS without vascular reconstruction. A paradigmatic case of potentially inoperable case is shown in Figure 1.

The “50–50” criteria was used to define posthepatectomy liver failure (PHLF),^15^ and clinical severity was classified according to the grades proposed by the International Study Group of Liver Surgery.^16^

Postoperative biliary fistula was defined as bilirubin concentration in drain discharge that exceeded 10 mg/dL for at least 3 days, starting from the 5th postoperative day. Ascites was defined by more than 10 mL/kg/day of drainage output from the abdomen after the 3rd postoperative day.^17^ Postoperative complications were stratified according to the Dindo–Clavien classification.^18^ Major complications (grade IIIb-IV) and operative mortality (grade V) were considered postoperative when they occurred within 1 month from surgery or during the hospital stay even when longer than 1 month.

Local tumor recurrence was defined as the cut-edge recurrence (true recurrence). Tumor recurrence in the “liver saved by M*b*C” was the recurrence in the liver parenchyma that would have been resected in the case of MH.

### Eligibility Criteria

**FIGURE 2:**
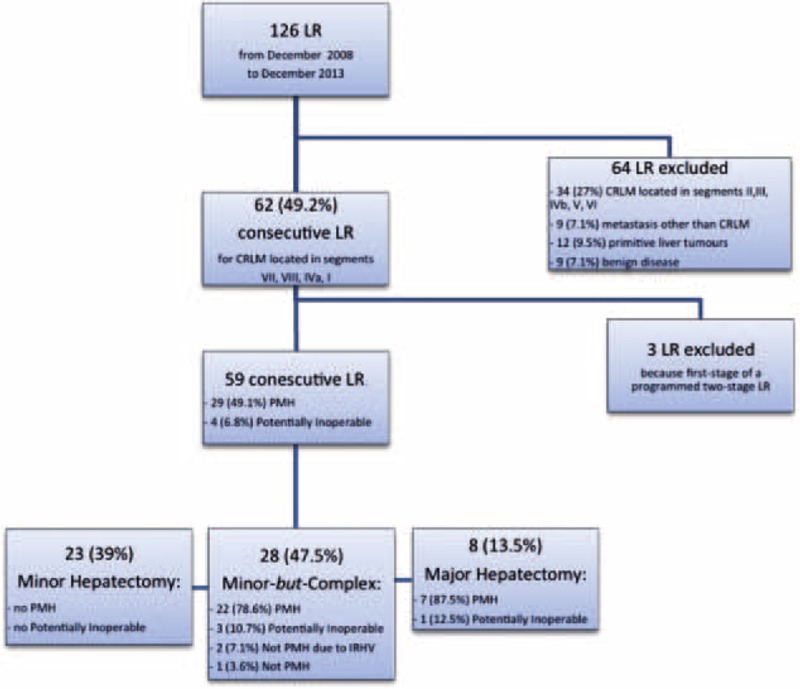
Flow-chart of all the liver resections performed from December 2008 until December 2013 with the selection of the study population. CRLM = colorectal liver metastasis; LR = liver resection; PMH = potentially major hepatectomy; IRHV = inferior right hepatic vein.

Among all consecutive patients who underwent LR in our Center from December 2008 until December 2013, those who had CRLM involving segments 1, 7, 8, or the cranial portion of segment 4 were included in this study (Figure 2). In particular, all patients potentially requiring an MH, independently from the previous treatment received, were enrolled. Only those patients undergoing a two-stage approach were excluded. This is a retrospective study conducted in accordance with the Declaration of Helsinki. All patients included provided written informed consent before treatment initiation, allowing physicians to administer the proposed treatment, perform the appropriate surgical intervention, and collect all the data in a site-specific database. None of the authors declare potential conflict of interest.

### Groups

According to the surgical procedure applied, the patients could be divided in three groups:

1. the MINOR resection group: removal of less than 3 adjacent segments without the need of IVC exposure and/or the main trunks of the HV;
2. the M*b*C resection group: removal of less than 3 adjacent segments but with the need of IVC exposure and/or the main trunks of the HV; and
3. the MAJOR resection group: removal of 3 or more adjacent segments.

### Preoperative Work-Up

Preoperative imaging work-up consisted in abdominal ultrasound, total-body CT, and magnetic resonance imaging. Patients were selected for surgery after a multidisciplinary board also involving medical oncologists and radiologists. The technical feasibility was established when residual liver volume with preserved blood inflow and outflow, and biliary drainage was expected to be more than 30% of the total functional liver volume. Liver volumes were calculated based on CT images.

### Operative Technique

A J-shaped laparotomy was usually performed. For those patients with tumor close to the CC a J-shaped thoracophrenolaparotomy was eventually carried out.^19^ For those patients with associated lesions of other organs the xipho-pubic incision was preferred. Laparoscopic approach was considered for small single lesions.

The IOUS was performed using a BK Medical – ProFocus Ultraview^®^.

**FIGURE 3:**
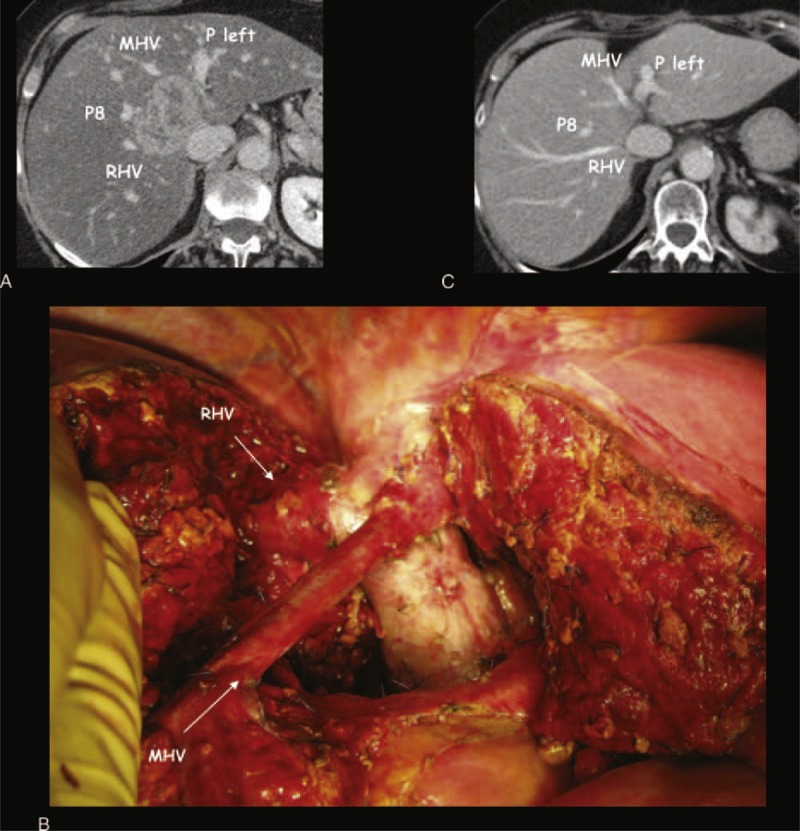
A case of minor-*but*-complex hepatic resection with hepatic vein preservation. This patient after 10 cycles of FOLFIRI and cetuximab was judged not operable with a major hepatectomy due to severe steatosis; therefore, she was treated with loco-regional treatment (transarterial chemoembolization). (A) Preoperative-CT scan shows the colorectal metastasis (CRLM) at the hepato-caval confluence (CC) under the plane of the hepatic veins and close to all the main vascular structures of the liver: right hepatic vein (RHV), middle hepatic vein (MHV), left portal branch (P left), anterior portal branch (P8), and cava vein. (B) This is the intraoperative field of the bisegmentectomy (I–IV) partially extended to the ventral part of segment VIII, with the exposure of all the main intrahepatic vascular structures; the intraoperative ultrasound excluded the infiltration of all the vascular structures in particular there was no vessel wall discontinuation for the MHV; the histological analysis showed 0 mm margins close to the main hepatic vessels, the liver cut surface was 212 cm^2^, the cumulative clamping time was 304 minutes, no blood transfusion was requested, the postoperative course was complicated by a transient elevation of creatinine, and the patient was discharged on the 17th postoperative day. (C) CT scan 18 months after liver surgery shows a normal right and left liver consistent with a parenchymal sparing surgery; the patient is alive and well without any sign of disease recurrence.

**FIGURE 4:**
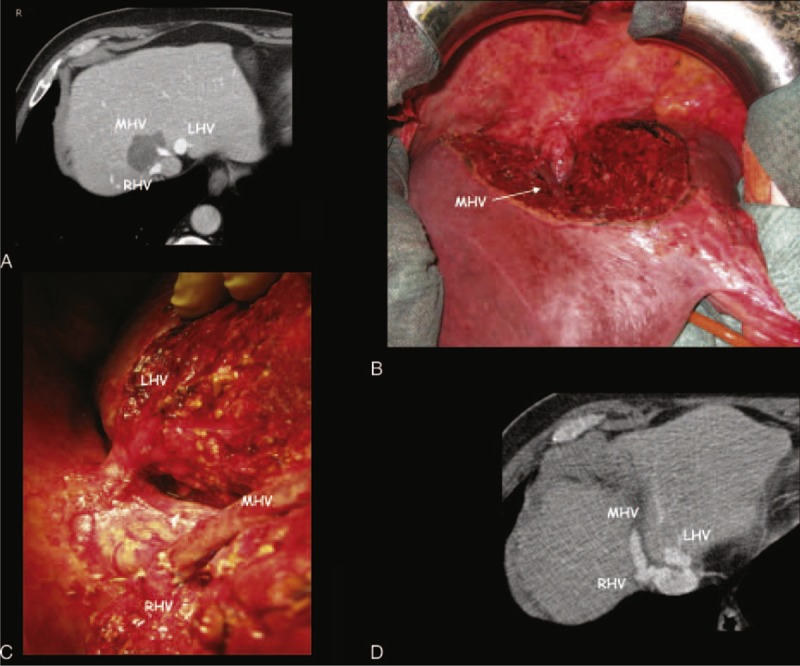
A case of minor-*but*-complex resection with hepatic vein anastomosis. This patient was judged not operable in another Center and was treated with loco-regional treatment (transarterial radio-embolization with yttrium). Due to a severe aortic valve stenosis 3 days before liver surgery, the patient was treated with percutaneous aortic valvuloplasty with reduction of gradient from 75 to 35 mmHg and started antiaggregant therapy. (A) Preoperative CT scan shows the colorectal metastasis (CRLM) at the hepato-caval confluence (CC) in contact with right hepatic vein (RHV) and apparently infiltrating the middle hepatic vein (MHV). (B) This is the intraoperative field of this minor-*but*-complex resection of segment 4a partially extended to segment VIII and to the paracaval portion of segment I, and with the exposure of all the main intrahepatic vascular structures at the CC: retrohepatic cava vein, left hepatic vein (LHV), and the termino-lateral anastomosis of the MHV with the RHV; the intraoperative ultrasound showed no MHV wall discontinuation, and the histological analysis confirmed the absence of MHV infiltration by the CRLM, the liver cut surface was 155 cm^2^, the cumulative clamping time was 185 minutes, no blood transfusion was requested, the postoperative course was uneventful, and the patient was discharged on the 6th postoperative day; 8 months after liver surgery, the patient presented a liver and lymph nodes recurrence; at the end of follow-up he is alive and well with disease recurrences. (C) A particular of the CC with the termino-lateral anastomosis between the MHV and the RHV. (D) The CT scan 4 months after liver resection shows the patency of the anastomosis between MHV and RHV.

Surgical strategy was based on IOUS findings regarding CRLM relationships with intrahepatic vascular structures. When the CRLM is in contact with the HV without any sign of wall discontinuation the attempt to dissect the vessel from the CRLM was always performed (Figure 3). If the vessel was resected the HV was always reconstructed by direct suture, or by anastomosis (Figure 4), or by the interposition of a ringed polytetrafluoroethylene (PTFE) 7 mm graft (Atrium Medical Corporation, NH) (Figure 1), except for the RHV in presence of IRHV.^13^

Since we have considered all the consecutive LRs performed from the beginning of our LR program, some technical aspects changed overtime to realize the M*b*C hepatectomy. The main changes are briefly reported in the following points.

The liver dissection at the beginning was performed by the crush-clamp technique or the ultrasonic dissector with the effort of the anesthesiologist to keep central venous pressure at less than 5 mmHg; hemostasis and biliostasis were achieved with extensive use of coagulation devices and the use of titanium clips to secure vessels >2 mm in thickness; and pedicle clamping was performed only in case of bleeding. With the increasing complexity of the resections, the titanium clips were abandoned and vessels >2 mm in thickness were ligated with thin (3/0) reabsorbable sutures (Vicryl®, Ethicon), bipolar electro cautery coagulation was used for <2 mm vessels only. Titanium clips and other coagulation devices were used only in laparoscopic liver surgery. Once liver transection was concluded, the liver cut surface was routinely monitored for about 50 minutes, and any bleeding or bile leakage points were controlled by thin reabsorbable sutures and bipolar forceps.

At the beginning of our LR experience, we preoperatively planned to treat single <1 cm CRLM by intraoperative thermoablation with the aim to spare liver parenchyma, but with the improvement of the surgical skill intraoperative thermoablation was no more used.

Another stepwise change in our study cohort regards the performing of liver dissection under intermittent pedicle clamping. At the beginning the Pringle manoeuvre was carried out for 15 minutes followed by 5 minutes of reperfusion. Then we prolonged the reperfusion time for 10 minutes. With the increasing complexity of the cases, especially for vascular reconstructions, longer clamping durations became inevitable, thus we adopted a reperfusion time equal to the clamping time.

### Outcome Measures

The primary objective of this study was to assess the rate of patients with liver CLRM candidate to MH who may benefit by the M*b*C resection approach. This was obtained by the difference between the rate of PMH plus potentially inoperable cases and the effective rate of the MH performed.

The secondary endpoint was to evaluate safety, complexity, and oncological results of the M*b*C approach. Taking into account that postoperative mortality is mainly related to the amount of resected liver^1^ and due to the limited number of patients in the MAJOR group, we focused on comparing these categories between patients treated with MINOR and M*b*C resections, who had comparable residual liver volume. For safety, we analyzed: rate of 0 mm margin resections, need of blood transfusion, hospital stay, rate of biliary leak, rate of postoperative ascites, rate of postoperative liver failure, and liver related deaths, need for continuous veno-venous hemofiltration (CVVH), morbidity and 90-day mortality using the Dindo–Clavien classification.^18^ For complexity, we analyzed: rate of previous liver treatment other then systemic chemotherapy, number of PMH, number of potentially inoperable cases, type of incision, duration of surgery, inflow clamping time, need of vascular reconstruction (and type of vascular reconstruction), occurrence of reconstructed vessels thrombosis, liver cut surface, and associated surgical procedures. For oncological results: rate of recurrence, site of recurrence, intrahepatic recurrence with rate of true local recurrence, and of recurrence in the “liver saved by the M*b*C approach”; overall survival (OS) and progression-free survival; rate of redo-surgery and rate of disease-free patients after the redo-surgery at the cut-off date.

### Patient Follow-up

After surgery, follow-up routine visits were scheduled every 2 months with physical examination, complete blood profile, CEA, and CT scan of the chest and abdomen (every 2 months for the 1st year, every 4 months for the 2nd and the 3rd year, and every 6 months thereafter). The cut-off date for analyses was June 1, 2014.

### Statistical Analysis

This is a retrospective cohort study. Continuous variables were compared using *t*-test, categorical variables were analyzed by Fisher exact test and Chi square test. Disease-free survival (DFS) and OS were determined using the Kaplan–Meier method and compared according to type of surgery using the log-rank test. Statistical significance was set at *P* < 0.05 for a two-tailed test. Statistical analyses were carried out using the statistical software package SPSS 19.0 (SPSS, Chicago, IL).

## RESULTS

### Primary Outcome

The General Surgery Unit of the Azienda Ospedaliero-Universitaria Pisana performs an average of 300 surgical procedures per year for the treatment of primitive colorectal neoplasms. The Liver Resection Program started in December 2008 when an experienced liver transplant surgeon^20,21^ entered in the colorectal surgical team with the aim to offer a complete surgical treatment opportunity for patients affected by colorectal malignancies.

From December 16, 2008 to December 31, 2013, 126 LRs were performed. Based on the aforementioned inclusion criteria, 62 consecutive LRs were included in this study. Three cases were excluded because they were the first stage of a programmed two-stage LR. The 59 consecutive LRs analyzed (Figure 2) were divided in 3 groups as follows:

1. MINOR: 23 (39.0%) LRs, in 1 (4.3%) case a <1 cm CRLM was intraoperative thermo ablated;
2. M*b*C: 28 (47.5%) LRs, in 2 (7.1%) cases a <1 cm CRLM was intraoperative thermo ablated; and
3. MAJOR: 8 (13.5%) LRs, in 2 (25%) cases a <1 cm CRLM was intraoperative thermo ablated.

Table 1 reports patients’ characteristics and oncological features of primary tumor and liver metastases. In the 32.1% of the M*b*C cases, the liver already received a previous treatment other than systemic chemotherapy, and in 20% it was a surgical treatment.

**TABLE 1:**
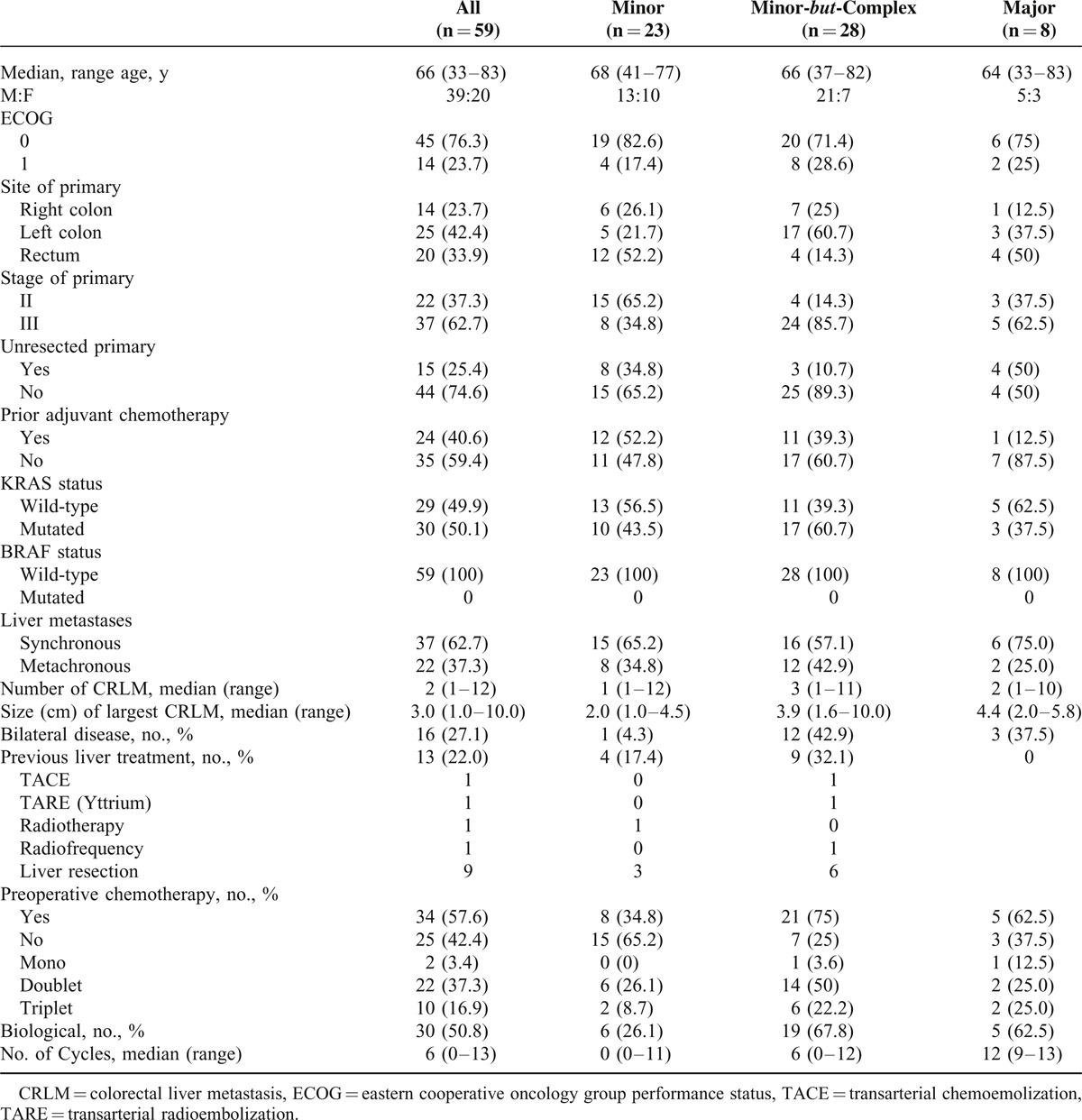
Baseline Patients’ Characteristics

At preoperative evaluation, 33 (55.9%) cases were deemed PMH (29, 49.1%) or potentially inoperable (4, 6.8%). Due to the implementation of the M*b*C approach the final number of MH performed was 8 (13.5%). The decrease of MH for CRLM located in segments 1, 7, 8, and the cranial portion of segment 4 was 42.4%.

### Secondary Outcome

The 90-day mortality was nil in the M*b*C resection group. Overall, only 2 (5.1%) patients died in the perioperative course: one because of myocardial infarction on the 9th postoperative day after a minor resection associated to rectal anterior resection, and another on the 32nd postoperative day due to septic hepatic artery bleeding after an MH associated to biliary reconstruction and retrohepatic caval resection.

We report in details in Tables 2–4 the differences in safety, complexity, and oncological outcomes between MINOR versus M*b*C and M*b*C versus MAJOR hepatectomies. Taking into account that postoperative mortality is mainly related to the amount of resected liver^1^ and given the small number of patients in the MAJOR group (8 cases), we here report the comparison of the secondary outcome measures between MINOR and M*b*C-resection groups, which had comparable residual liver volume.

**TABLE 2:**
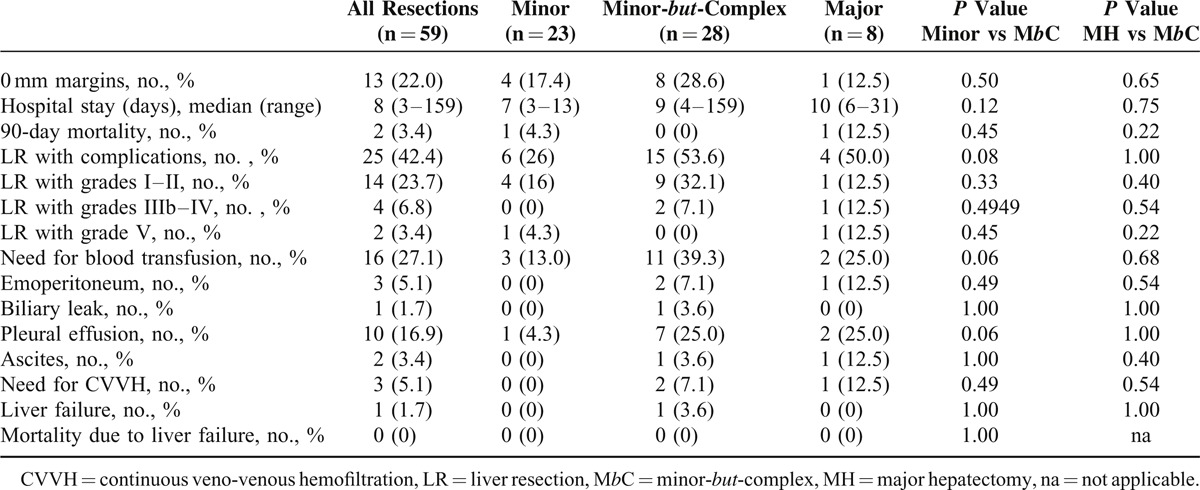
Safety of the Minor-*but*-Complex hepatectomy

**TABLE 3:**
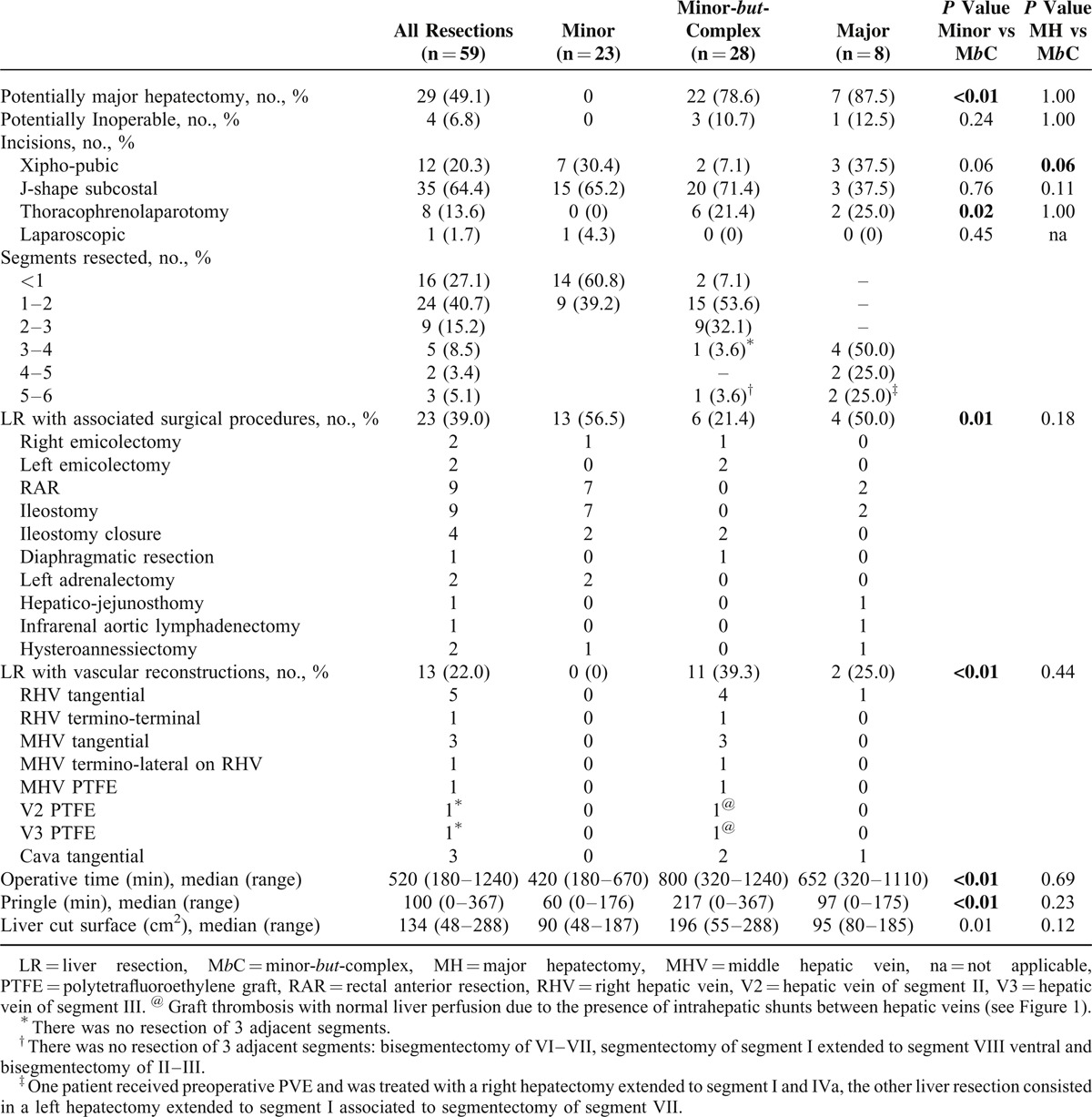
Complexity of the Minor-*but*-Complex Hepatectomy; Significant Values Are Presented in Bold

**TABLE 4:**
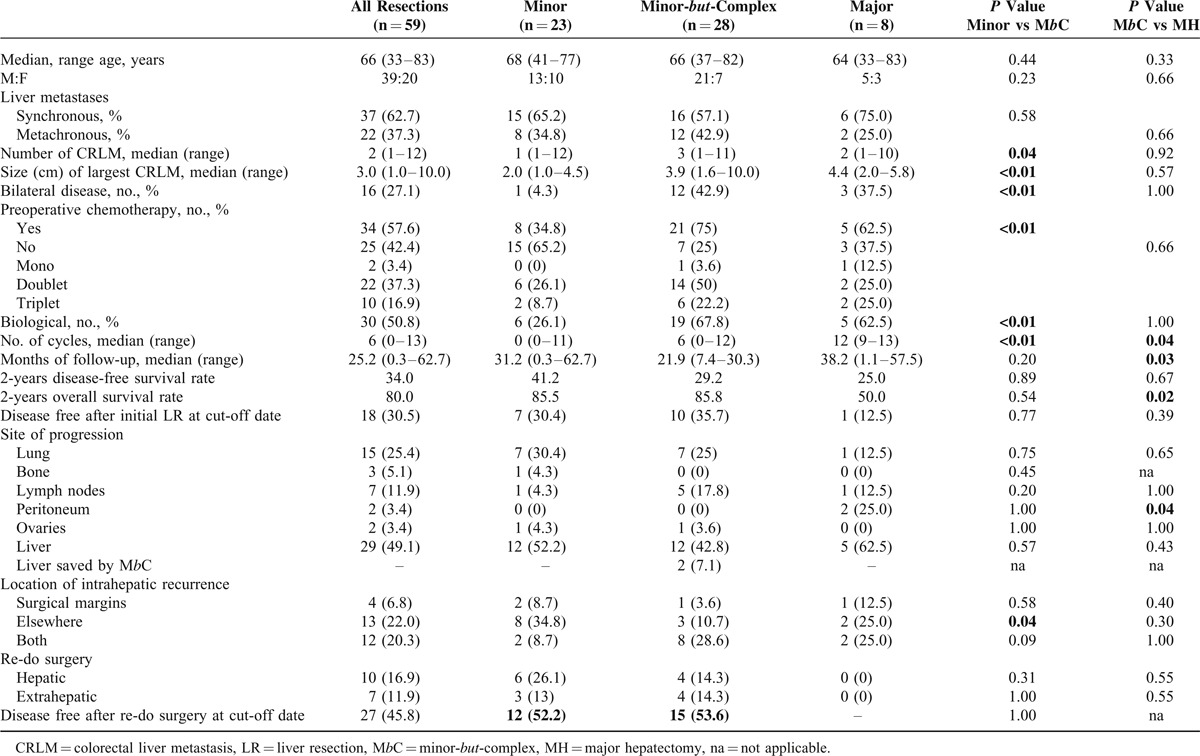
Patients’ Characteristics and Oncological Results of the Minor-*but*-Complex Hepatectomy, Significant Values Are Presented in Bold

Regarding safety (Table 2), 1 case (3.6%) of severe but not fatal PHLF was observed in the M*b*C group. This patient was 1 of the 2 cases with severe hypotension during M*b*C resection associated with postoperative multiorgan dysfunction and sepsis by KPC-producing *Klebsiella pneumoniae*. One of them was reoperated twice because of hemoperitoneum and both the patients needed CVVH. During the follow-up all the livers were well perfused, PTFE graft thrombosis occurred in 2 out of 3 grafts, but only when intrahepatic shunts between main HV were present (Figure 1).

Table 3 describes the results for complexity. In the M*b*C group 2 patients had the resection of more than 3 segments, but there were no 3 adjacent segments resected. In the M*b*C group, the 89.3% of LRs were PMH or potentially not operable. In the M*b*C group, there was a significant higher use (*P* = 0.02) of the thoraco-abdominal approach (21.4%).

In the 28 M*b*C resections, the parenchyma was spared using different approaches: in 11 (39.1%) cases using a vascular reconstruction, in 2 (7.1%) cases resecting the RHV at the CC in presence of an IRHV, and in 15 (53.6%) cases by the complete exposure of the HV (as shown in Figure 3). In these cases median operative time (800 minutes, *P* < 0.01), median Pringle time (217 minutes; *P* < 0.01), and median liver cut surface (196 cm^2^; *P* = 0.01) were significantly higher than the MINOR resection group, where there was a significant higher incidence (*P* = 0.01) of associated surgical procedures (56.5%).

Table 4 summarizes the oncological results. The median follow-up in the groups of MINOR and M*b*C resections was 22.2 (0.3–62.7) months with no significant difference between them. Patients in the M*b*C group were more complex also from the oncological point of view. The median number of 3 CRLM and the median size of the largest CRLM (3.9 cm) were significantly higher than in the MINOR group (*P* = 0.04 and *P* < 0.01). As a consequence, more patients (75%) received preoperative chemotherapy with a median number of 6 cycles (*P* < 0.01).

There was no significant difference between MINOR and M*b*C groups in DFS (41.2% versus 29.2%, *P* = 0.89) and OS (85.5% versus 85.8%, *P* = 0.54). There was a statistically significant difference in OS between M*b*C and MAJOR hepatectomies (85.8% versus 50.0%, *P* = 0.02), but not in the DFS (29.2% versus 25.0%, *P* = 0.67). One third of the M*b*C patients had a second hepatic or extrahepatic surgery, which increased the DFS at the cut-off date from 35.7% (after the first LR) to 53.6%.

## DISCUSSION

In the recent years, the availability of more pharmacological options and the evolution of the interventional techniques increased complexity of metastatic colorectal cancer treatment. In order to maximize patient's survival, a multidisciplinary management is mandatory, and the surgical approach should balance the achievement of the radical aim with the possibility of reoperation and the need to perform pre- and postoperative systemic treatments. In this context, we reported our experience with M*b*C resections that allowed for resecting patients otherwise inoperable or candidate to MH, which is associated with high mortality and major morbidity. In this retrospective study, we showed that the safety profile of M*b*C resections is comparable to that of minor resections, in spite of the higher degree of complexity due to the spread of the tumor and to the prior treatments applied. Nevertheless, this novel operative technique involved multiple combined procedures (including the surgical management of intrahepatic vessels) prolonging the operative times as well as the clamping times. However, it has already been shown^22,23^ that long operations were associated with those series featuring the best results in terms of safety. Indeed, in our M*b*C resections, the median hospital stay was 9 days (7 days in patients with MINOR resections) and the rate of major complications (7.1%) was not higher than the 17% to 26% reported in other series of noncirrhotic patients.^23,24^ Among the factors that may explain these favorable results and the surprisingly 0% perioperative mortality, there is the large amount of residual parenchymal volume left after the resections.^1^

In the last 2 decades, other multistep approaches (ie, PVE, two-stage resection) attempted to increase the liver mass left after resections. Nevertheless, 15% of the patients did not benefit from PVE, mainly because of disease progression or insufficient remnant liver hypertrophy,^25^ thus up to 33% of patients did not complete the two-stage strategy.^26^ Although the interval between the 2 steps may help to identify patients with better prognosis by revealing tumor biology behavior,^26^ it is not clear yet whether tumor progression could be favored by the growth stimuli of the induced liver cell hyperplasia.^6^ The recent introduction of the ALPPS technique allows for a significant contraction of interval between the 2 steps, but the potential oncological effect of the massive regeneration remains an open issue.^7^

Similarly to what reported with the radical but conservative parenchymal-sparing surgery,^27,28^ the M*b*C approach provided instead the opportunity to treat in a single-step procedure complex cases with 0% mortality at 90 days. This is remarkable when compared with the high perioperative mortality of MH. In our study cohort, only 8 patients underwent MH and 1 died (12.5%), which is in line with the 10.3% perioperative mortality reported by Cauchy et al,^29^ when MH was performed in initially unresectable patients. Noteworthy, in these complex cases Brouquet et al^26^ reported a high postoperative mortality (2% and 6% at 30 and 90 days) also for the two-stage approach, as well as Schnitzbauer et al^6^ reported a 44% grade III and IV complications with 12% hospital lethality in the first ALPPS series.

Overall, in our cohort of 59 consecutive patients with CRLM localized in segments 7, 8, 4a, and 1, the development of the M*b*C resection approach lowered by 42.4% the need for MH and opened the way to a new extensive parenchymal sparing liver surgery. To perform such a complex surgery the liver dissection technique changed stepwise, abandoning the coagulation devices to better preserve the liver skeleton. A major effort was requested to the anesthesiology team who developed a specific expertise in avoiding hypotension during the very long intermittent clamping times. In fact, at the beginning of our series, the extensive use of pedicle clamping was associated with intraoperative hypotension and followed by multiorgan failure in 2 patients, including severe liver failure in 1 case. In our initial experience, we also reported the 367 minutes longest cumulative clamping time that, however, was not associated to an unfavorable outcome.^30^ Indeed, none of the patients died after an M*b*C resection in spite of the complexity of the surgery, who is underlined by the extension of the liver cut surface, by the 32.1% of previous treatments other than systemic chemotherapy, and by the 39.3% of LRs with vascular reconstruction.

Vascular reconstructions were reported to increase the mortality risk when are associated to MH,^29,31^ but this observation is not confirmed by all the authors.^32,33^ In the M*b*C setting, surgical management of hepatic vascular structures did not represent an additional risk factor for postoperative liver failure. We may explain this result taking into account that the HV reconstruction in our approach is not done on the single HV left, but it is set to work as “a bridge” until intrahepatic shunts maturation. The residual liver perfusion obtained with our approach is optimal and comparable to that obtained by Torzilli et al,^8^ which requires real-time hemodynamic IOUS study to identify the liver skeleton and a special skill to transform the bi-dimensional view of the IOUS in the 3-dimensionality of the liver cut surface. Thus, our technique may overcome an obstacle to a wider diffusion among surgeons of the parenchymal sparing concepts introduced by Torzilli et al. The M*b*C approach is aimed to maintain the “vertical” blood flow in main HV as “a bridge” to spontaneous maturation of “horizontal” blood flow in peripheral intrahepatic shunts. The article by Hwang et al^34^ reinforced our concept of “the bridge” solution. In fact, the authors, describing the role of ringed PTFE grafts for the reconstruction of MHV in living donor right liver transplantation, documented that the progressive obliteration of the PTFE graft does not affect the liver graft perfusion and survival. A recent study by Hribernik and Trotovšek^35^ demonstrated the existence of peripheral intrahepatic venous anastomoses of 1 mm of diameter or less in the corrosion casts obtained from macroscopically normal cadaveric livers. These thin communicating veins are undetectable in condition of normal blood flow, even with new-generation CT, or magnetic resonance imaging.^9^ The case described in Figure 1 is highly explicative of the M*b*C approach. In this potentially inoperable patient, the left outflow was completely reconstructed using 3 ringed PTFE grafts. The CT scan at 5 months from LR shows the maturation of the shunts between V3 (hepatic vein for segment 3) and MHV, which were not evident on the CT scan at postoperative day 11. For this reason the left liver remained well perfused in spite of the thrombosis in 2 PTFE grafts.

The other methods used to maintain the blood flow in main HV at the CC alternative to perform an MH are the preservation of the HV with 0 mm margin (as described in Figure 3), the tangential resection and direct suture of the HV, or the direct anastomoses between main HV (Figure 4).

In conclusion, M*b*C LRs represent a new way to pursue a parenchymal sparing policy in liver surgery. Liver transplantation surgical techniques may be used to transform an MH in a minor hepatectomy respecting the liver skeleton, which allows for the spontaneous maturation of intrahepatic shunts between main HV. M*b*C hepatectomies are more demanding than standard resections, but more affordable without special IOUS expertise because vessels reconstructions avoid the complexity of ultrasound identification and preservation of intrahepatic shunts.

Overall, the M*b*C approach allowed treatment of potentially inoperable cases and reduced the need for MHs without negative impact on safety and oncological outcomes. Despite a more advanced disease at presentation and a different therapeutic algorithm, M*b*C patients achieved a good oncologic outcome comparable with patients treated with a MINOR LR. These results support further efforts in the multidisciplinary management of patients affected by metastatic colorectal cancer.

## UNCITED REFERENCE

^30^
